# Up-bottom assessments of nutrient supply and gaseous pollutant from Chinese wheat straw field management

**DOI:** 10.1038/s41597-024-03151-0

**Published:** 2024-04-03

**Authors:** Dongxue Li, Jun Gu, Xiaoqin Chen, Yiliu Wang, Dianjun Lu, Solomon Yokamo, Huoyan Wang, Peng Hou

**Affiliations:** 1 State Key Laboratory of Soil and Sustainable Agriculture, Institute of Soil Science, Chinese Academy of Sciences, Nanjing, 210008 China; 2 College of Modern Agricultural Sciences, Chinese Academy of Sciences, Beijing, 100049 China; 3 Institute of Crop Sciences/Key Laboratory of Crop Physiology and Ecology, Chinese Academy of Agricultural Sciences, Ministry of Agriculture and Rural Affairs, Beijing, 100081 China

**Keywords:** Environmental impact, Environmental monitoring

## Abstract

To achieve resource efficiency, and carbon neutrality, it is vital to evaluate nutrient supply and gaseous pollutant emissions associated with field management of bio-straw resources. Previous straw yield estimates have typically relied on a constant grain-to-straw yield ratio without accounting for grain yield levels in a given region. Addressing this high-resolution data gap, our study introduces a novel empirical model for quantifying grain-to-straw yield, which has been used to gauge wheat straw field management practices at the city level during 2011–2015. Utilizing both statistical review and GIS-based methods, average nitrogen (N), phosphorus (P), and potassium (K) supplies from straw field management stood at 1510, 1229, and 61700 tons, respectively. Average emissions of PM_2.5_, SO_2_, NOx, NH_3_, CH_4_, and CO_2_ due to straw burning were 367, 41, 160, 18, 165, and 70,644 tons, respectively. We also reported uncertainty from Monte Carlo model as the 5th-95th percentiles of estimated nutrient supply and gaseous pollutant. These insights will provide foundational support for the sustainable and environmentally friendly management of wheat straw in China.

## Background & Summary

Growing global food demand has spurred rapid advances in cereal crop production over recent decades, concomitantly leading to significant crop straw production^1,2^. Recognized as a vital renewable energy source, straw has garnered considerable attention both from agricultural perspectives and in terms of environmental emissions^3–6^. Recycling straw is perceived as a proficient resource management strategy in agriculture, boasting potential benefits such as enhanced soil quality and increased crop yield^7,8^. Conversely, the practice of open straw burning poses a grave threat by emitting particulates and gaseous pollutants that can create haze and pollute the air, a pressing issue particularly in developing nations^9–11^. In China, a leading straw producer, the proportion of recycled straw has yet to surpass 60% at present, even amidst stringent straw burning bans since 2010^12^. Hence, it is necessary to accurately characterize straw field management to ensure efficient resource utilization, optimize agricultural productivity, and mitigate environmental repercussions.

A high-resolution agricultural nutrient supply potential and gaseous pollutant emissions caused by field management of crop straw is necessary for discipline researchers and policymakers to assess and optimize straw resources. However, current studies may provide limited insights into the relevant resources’ researches and policy implications for the following limitations. First, typical straw yield estimations hinge on a constant the grain-to-straw ratio (the proportion of grains yield to straw yield) without accommodating grain yield levels in a given region^13,14^. The meta-data study in intensive wheat production indicated that the increase in wheat grain yield in the recent decades has been attributed to the simultaneous enhancement in both total dry matter accumulation and dry grain to straw ratio in China^15^. Moreover, there were considerable variations in wheat grain yield and grain to straw dry matter ratio across different ecological wheat planting zones^16^. Hence, the nutrient supply and gaseous pollutant from various straw field management methods have vague estimates at the regional and national level, which has a vital role in ensuring food security, balanced fertilization, and supporting the prosperity of agricultural green development^17–19^. Furthermore, most previous studies have had a narrow focus on singular aspects of straw resource management such as straw recycling and open straw burning^10,20,21^. Few studies provide the specific field managements of crop straw, especially combined with high-resolution maps at the regional level for individual crops.

**Fig. 1:**
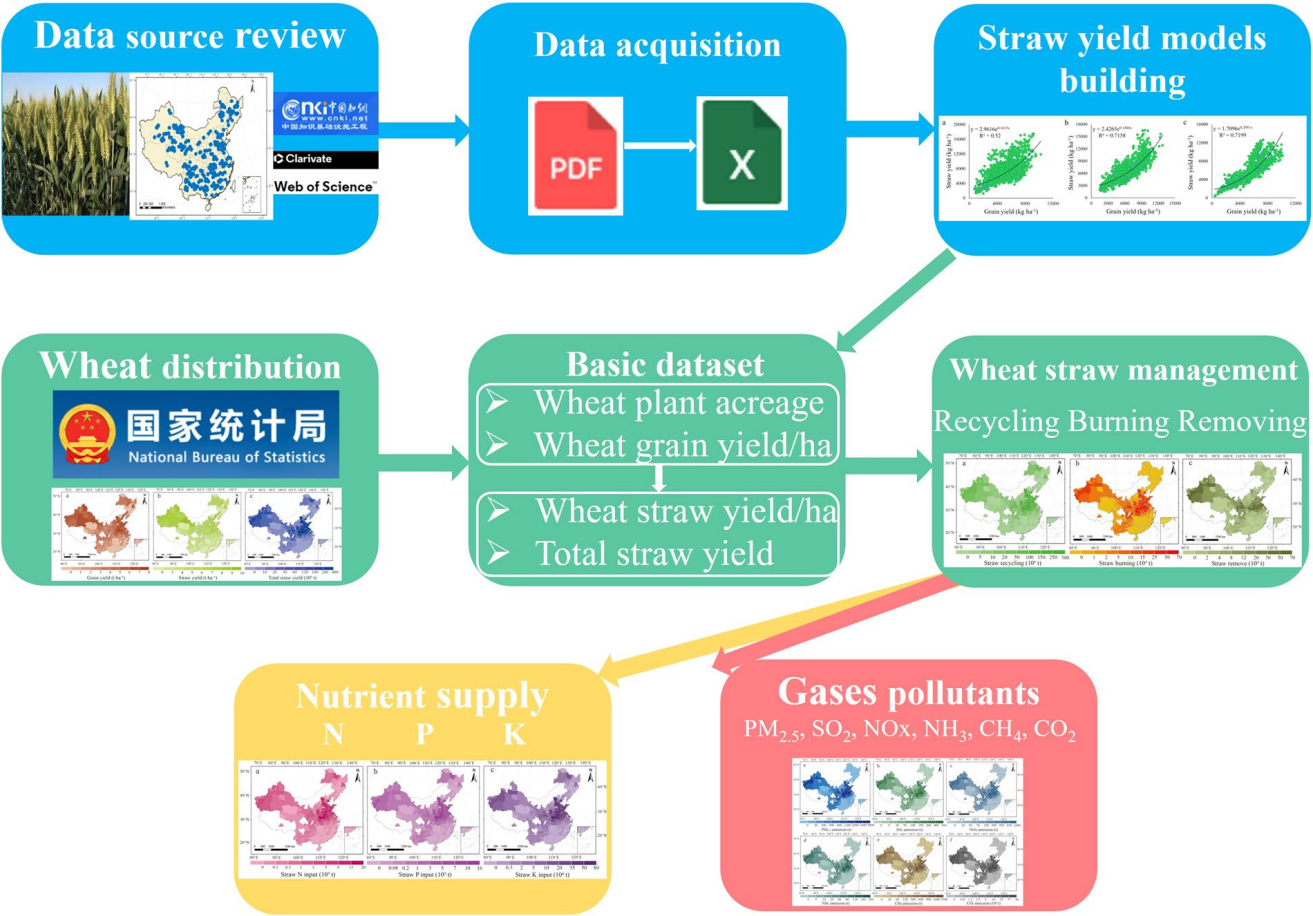
Framework illustrating the nutrient supply and gaseous pollutants from Chinese wheat straw field management.

In response to these challenges, we developed a novel empirical regional straw yield estimation method that captures crop straw resources in mainland China; using it, we created a high-spatial-resolution dataset across Chinese main wheat planting zones (Fig. 1). Given the pronounced wheat straw yield disparities in Chinese wheat crops, we zeroed in on the field management patterns of straw and the emissions from straw burning. Combining statistical and GIS-based methods, we assembled a city-level dataset detailing straw field management, including present-day wheat outputs and field management practices. Additionally, our dataset includes nutrient supplies (N, P, and K) and gaseous pollutant emissions (PM_2.5_, SO_2_, NOx, NH_3_, CH_4_, CO_2_) stemming from straw management. Collectively, our findings illuminate wheat straw resource field management in agricultural operations and provide granular insights for spatial-level environmental impact assessments in China.

## Methods

### Data collection

**Fig. 2:**
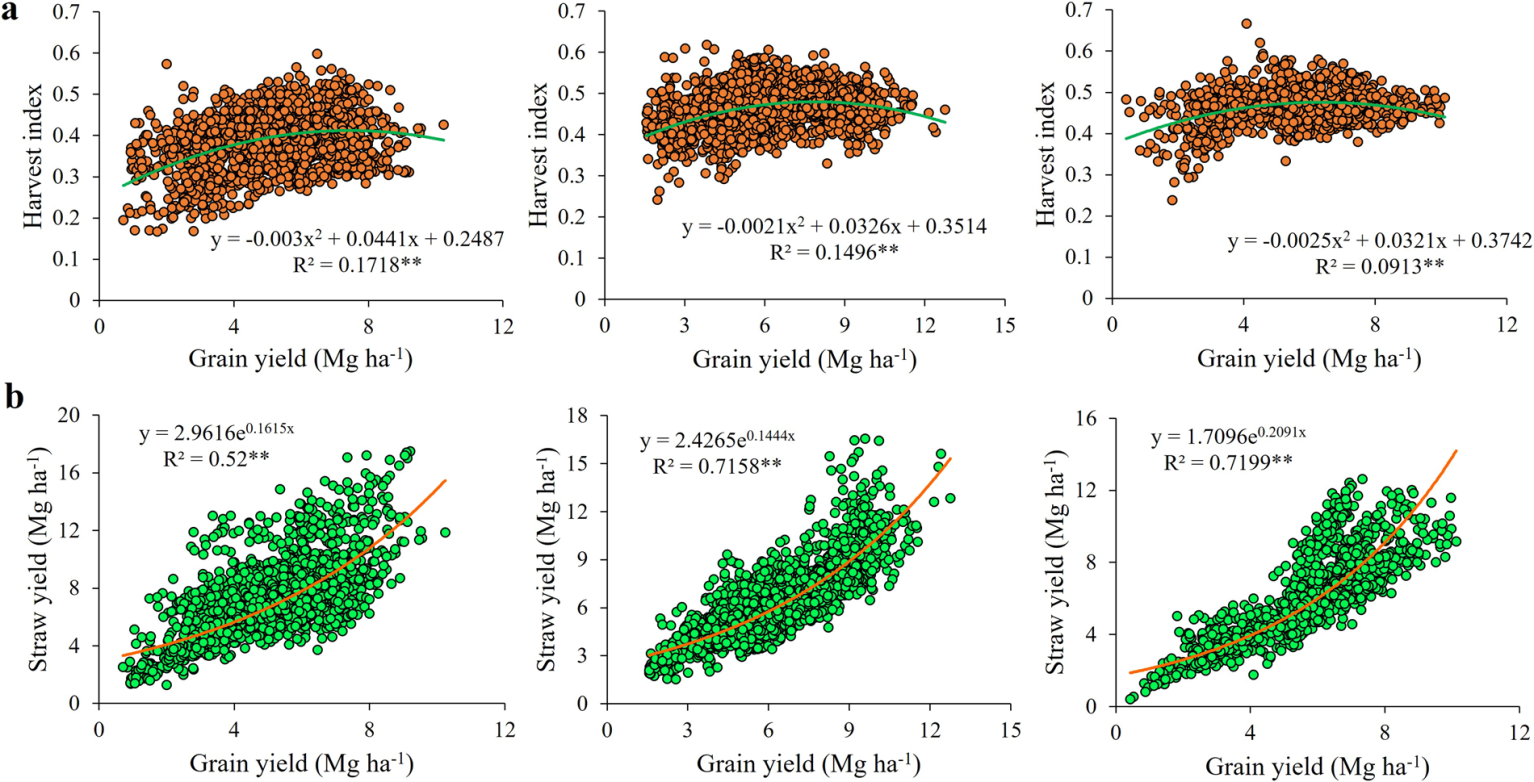
Wheat harvest index (**a**) and straw yield (**b**) in correlation with grain yield for northern China, central China, and southern China, respectively.

To elucidate straw yields across diverse regions of China, we instituted a novel empirical model, drawing upon 1728, 2209, and 1184 observations [grain yield, straw yield, and harvest index (the proportion of grains yield to total biomass)] after 2000 for northern China (NC), central China (CC), and southern China (SC), respectively (Fig. 2). The NC, CC, and SC data were sourced from 60, 42, and 10 peer-reviewed articles identified via the Web of Science (http://apps.webofknowledge.com/) and the China Knowledge Resource Integrated Database (http://www.cnki.net/) (see Supplement). Bibliography Retrieval was conducted with the key words “wheat”, “straw yield or harvest index”, and “north of China” “central of China”, or “south of China”. To bolster accuracy and curtail bias, we incorporated the following inclusion criteria for article selection: (1) experiments were field-based; (2) they were conducted in regions of NC such as Shanxi, Shaanxi, Ningxia, Gansu, Qinghai, and Xinjiang, CC such as Beijing, Hebei, Henan, and Shandong, and SC such as Anhui, Jiangsu, Zhejiang, Hubei, Chongqing, Sichuan, Guizhou, and Yunnan; and (3) the publications provided accessible data on wheat grain yield, straw yield, or the harvest index.

The production and distribution of total biomass to grain may be simultaneously restricted during the transition from low to middle yield^15^, due to inadequate nutrient availability (nitrogen, phosphate, and potassium) as well as improper crop management such as delayed sowing dates. When grain yield further increased with favorable nutrient environment and desirable crop management, the distribution of dry matter to the grain may be further stabilized or even reduced while the total biomass was the main reason of grain yield increases. Improved tiller population density resulted in severe competition for nutrient, water, light in the tiller population, thereby depressing the biomass allocation into spike^22,23^. As a result, as grain production increased further, more dry matter could be assigned to the straw; the exponential function should be more reasonable to depict the relationship between grain yield and straw yield, while the harvest index had reached stable or even reduced with grain yield increase (Fig. 2b).

### Dataset preparation

To assess nutrient supply potential and gaseous pollutant emissions from various wheat straw field management (straw recycling, straw burning, and straw removing), we developed a high spatial resolution dataset of wheat straw resources at the city-level from 2011 to 2015 in mainland China. Straw recycling refers to the recycling to the fields by crushing, ploughing, and mulching. Straw removing refers to the removal of straw from fields for other uses.

Initially, data on wheat grain yield per hectare and wheat planting acreage from 2011 to 2015 were amassed from the National Bureau of Statistics (http://www.stats.gov.cn/sj/) (Table S1). The per hectare yield of straw was derived using the Wheat Straw Yield Empirical Model. The wheat total straw yield at the city-level was calculated as per Eq. (1):

1 $${TY}_{s,i}={PY}_{s,i}\times {Area}_{i}$$

where *TY*_*s,i*_ and *PY*_*s,i*_ are the total straw yield in each city and straw yield per hectare, respectively; *Area*_*i*_ is the planting area of wheat; and *i* (1, 2, 3…m) is the city.

Subsequently, we quantified the amounts of straw management in the field, whether via recycling, burning, or removing. The proportional data regarding these field management modes were extracted from the published scholarly journals^24^. Therefore, the amounts of straw for these modes were computed using Eq. (2):

2 $${AS}_{d,i}={TY}_{s,i}\times {PS}_{d,i}$$

where *AS*_*d,i*_ and *PS*_*d,i*_ are the amounts of various straw disposed and the proportional data regarding field management models (see Supplement Table S2).

Next, we evaluated the potential nutrient supply to croplands via the recycling and burning of straw in the field. We calculated the total N, P, and K nutrient supplies using Eq. (3):

3 $${TNS}_{i,j}=\left({AS}_{d,i,r}+{AS}_{d,i,b}\right)\times {NC}_{i,j}$$

where *TNS*_*i,j*_ is the total N, P, and K supplies into the field; *AS*_*d,i,r*_ and *AS*_*d,i,b*_ are the amounts of straw recycled and burned, respectively; *NC*_*i,j*_ is a straw nutrient concentration parameter in which N, P, and K are 0.49%, 0.32%, and 18.01% for NC, 0.52%, 0.37%, and 18.09% for CC, and 0.51%, 0.32%, and 18.01% for SC, respectively^25^; and *j* (1, 2, 3) is the type of nutrient (N, P, or K).

Finally, emissions of gaseous pollutants (PM_2.5_, SO_2_, NOx, NH_3_, CH_4_, CO_2_) resulting from open straw burning were assessed using emissions coefficients calculated using Eq. (4):

4 $${GP}_{i,k}={AS}_{d,i,b}\times {EC}_{k}$$

where *GP*_*i,k*_ is the emissions from open straw burning; *EC*_*k*_ is the emissions coefficient of gaseous pollutants in which PM_2.5_, SO_2_, NOx, NH_3_, CH_4_, and CO_2_ are 7.6 g kg^−1^, 0.85 g kg^−1^, 3.3 g kg^−1^, 0.37 g kg^−1^, 3.4 g kg^−1^, and 1460 g kg^−1^, respectively^26^; and *k* (1, 2, 3…m) is the type of gaseous pollutant (PM_2.5_, SO_2_, NOx, NH_3_, CH_4_, CO_2_).

### Data management

Data were visualized using Microsoft Office Excel 2016 (Microsoft Corp, Redmond, WA, USA). Graphs were made using both Microsoft Office Excel 2016 and PowerPoint 2016. The uncertainty analysis was generated in R version 3.3.1 (R Core Team 2016). Map vector layers were sourced from the Resource and Environment Data Cloud Platform and the National Catalogue Service for Geographic Information, with all mapping done using ArcGIS 10.2 software.

## Data Records

**Fig. 3:**
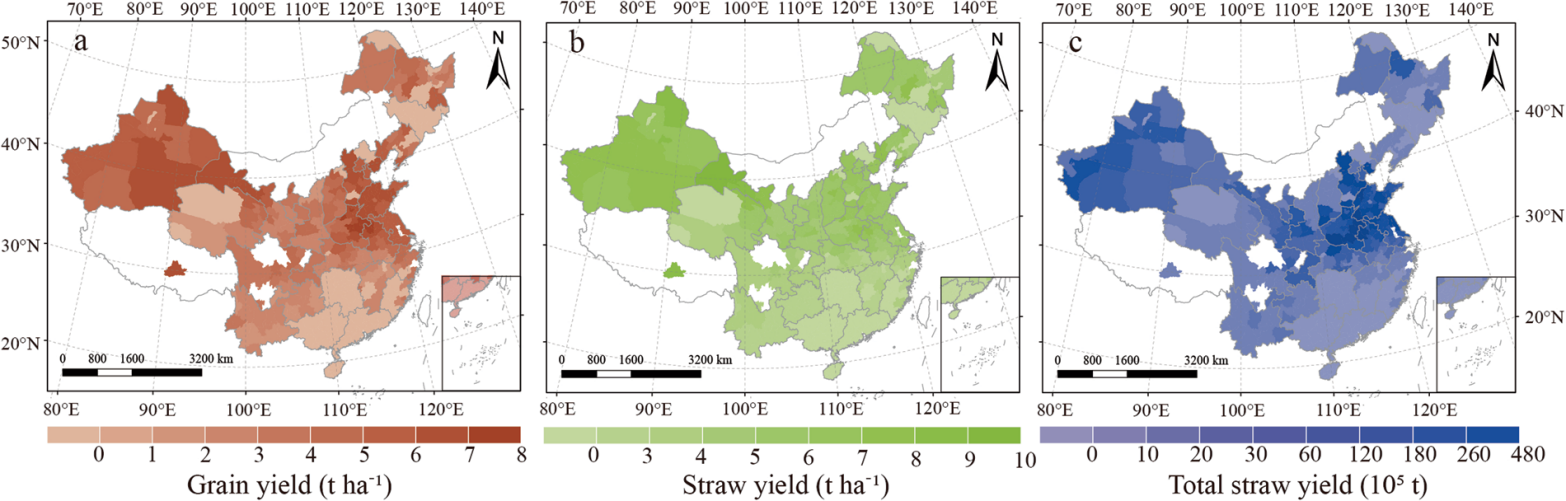
Spatial representation of wheat grain yield (**a**), wheat straw yield (**b**), and total wheat straw yield (**c**) on a city-by-city basis in China from 2011–2015. The values in the figures are the average amount from 2011 to 2015. White colors indicate no available data for the current estimates.

**Fig. 4:**
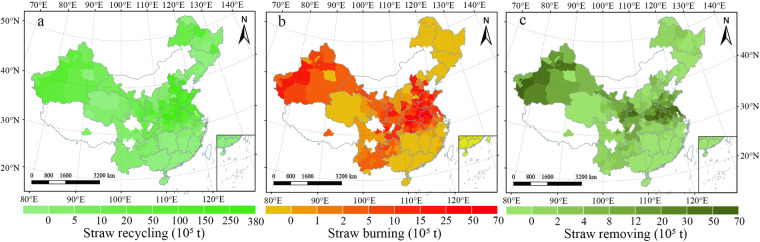
City-level spatial distribution of total wheat straw yield by field management method in China from 2011–2015. Modes include (**a**) straw recycling, (**b**) straw burning, and (**c**) straw removing. The values in the figures are the average amount from 2011 to 2015. White colors indicate no available data for the current estimates.

**Fig. 5:**
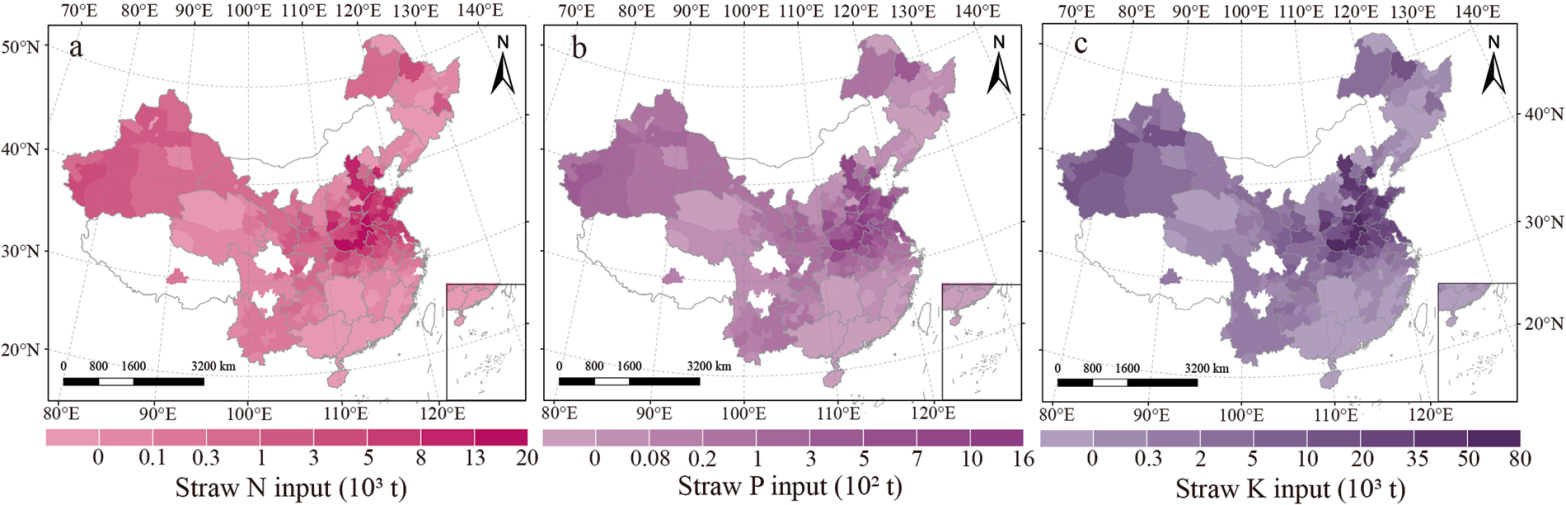
City-level spatial distribution of nutrient supply from straw recycling and burning, detailing (**a**) nitrogen (N), (**b**) phosphorus (P), and (**c**) potassium (K) from 2011–2015 in China. The values in the figures are the average amount from 2011 to 2015. White colors indicate no available data for the current estimates.

**Fig. 6:**
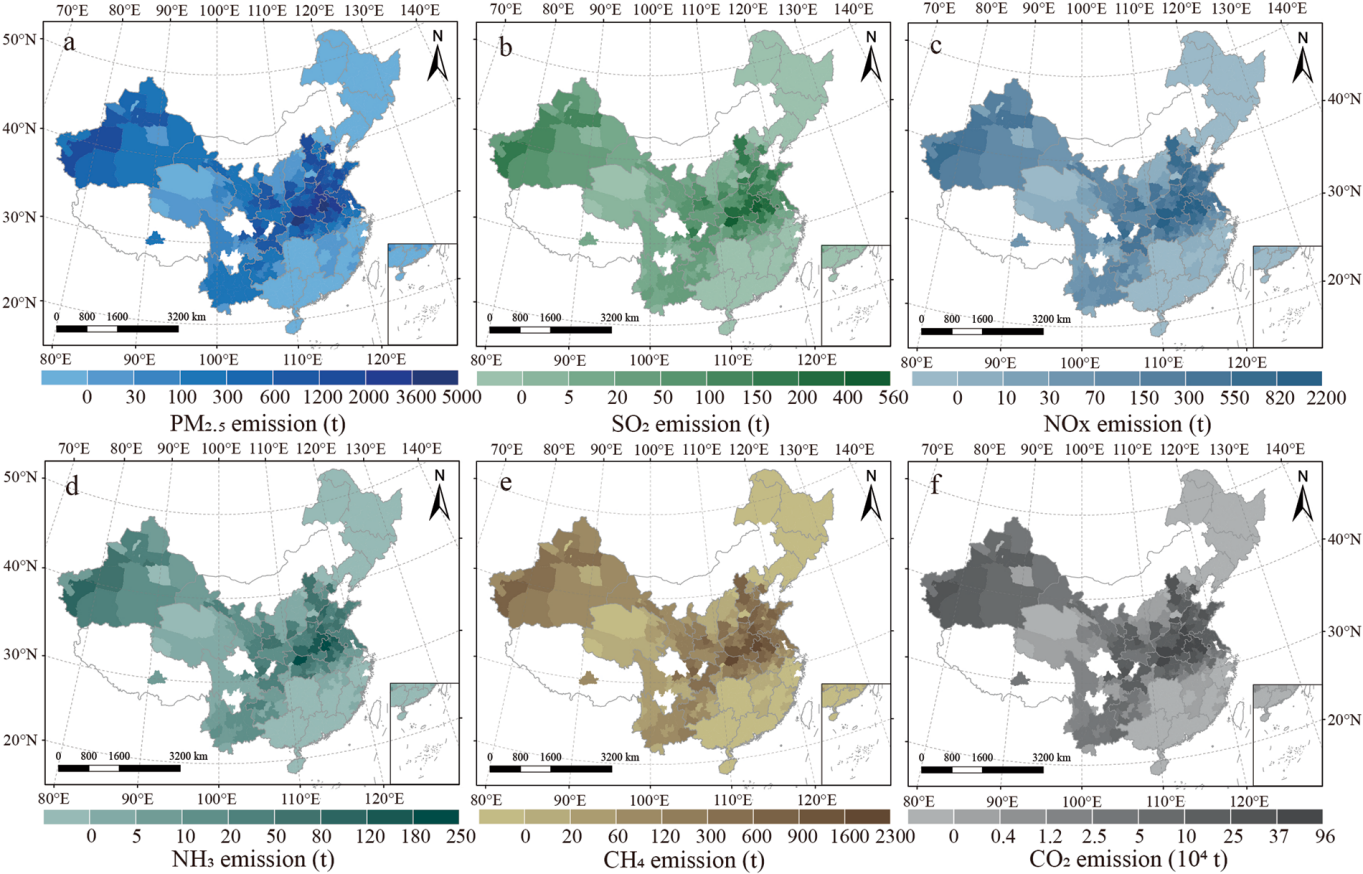
Spatial visualization of gaseous pollutants resulting from straw burning at the city level in China from 2011–2015. Emissions include (**a**) PM_2.5_, (**b**) SO_2_, (**c**) NOx, (**d**) NH_3_, (**e**) CH_4_, and (**f**) CO_2_. The values in the figures are the average amount from 2011 to 2015. White colors indicate no available data for the current estimates.

The dataset is available at the National Tibetan Plateau Data Center^27^; they are compatible with ArcGIS. Our datasets were exhibited in Excel file format with the following five sheets: “models building”, “wheat planting situation”, “straw field management”, “nutrient supplies”, and “gaseous pollutants”. The first sheet recorded wheat grain yield (unit: kg ha^−1^) and straw yield (unit: kg ha^−1^) of Chinese various regions (Fig. 2). The second sheet described the wheat planting acreage (unit: 1000 ha), grain yield (unit: kg ha^−1^), straw yield (unit: kg ha^−1^), and total straw yield (unit: 10000 tons) of each city, respectively (Fig. 3). The third sheet contained the total straw recycling amount (unit: 10000 tons), total straw burning amount (unit: 10000 tons), and total straw removing amount (unit: 10000 tons) of each city, respectively (Fig. 4). The fourth sheet consisted of the N supply (unit: tons), P supply (unit: tons), and K supply (unit: tons) from straw recycling and burning in the field of each city, respectively (Fig. 5). The last sheet was composed of PM_2.5_ (unit: tons), SO_2_ (unit: tons), NOx (unit: tons), NH_3_ (unit: tons), CH_4_ (unit: tons), and CO_2_ (unit: tons) emissions from open-field straw burning of each city, respectively (Fig. 6). “0” and “NA” fields indicated municipal districts and cities without planting wheat and without available data.

Based on the 5-year average of data the national total wheat straw amount was 15.94 Mt, contributing 19.7% of the national total straw amount (81.14 Mt). Moreover, the national CO_2_ and CH_4_ emissions from wheat straw burning were 23.27 Mt and 0.054 Mt; contributing 21% of national total CO_2_ emissions (112.8 Mt) and 30% of national total CH_4_ emissions (0.178 Mt) in China^28^. Regional characteristics of gaseous pollutants and nutrient supply are closely related to regional straw yield, straw recycling rate, and straw burning rate. In general, the central region was highest followed by the northwest region.

## Technical Validation

### Uncertainty analysis

This study used a large data sample to assess wheat straw field management in China, with a particular focus on clarifying the spatial distribution of nutrient supplies potential and gaseous pollutants emission. We evaluated uncertainty in the dataset is mainly related to the data reliability and representativeness, straw nutrient concentrations, and emissions factors through Monte-Carlo simulation. First, the newly wheat straw yield empirical model was determined by 5121 observations from field experiment, which should simultaneously contain wheat yield and straw yield or harvest index throughout China’s croplands. The data were generated by our team and colleagues, as mentioned above, and had been rigorously screened, quality-checked, and published in eminent international journals (see Supplement). All remaining observations were sourced from authoritative repositories, as mentioned above, notably the Web of Science and the China Knowledge Resource Integrated Database (see Supplement). Second, the quality control of all results is significantly dependent on the collection of the wheat grain yield per hectare and wheat planting acreage, which were obtained from the National Bureau of Statistics. Third, data related to straw field management, nutrient concentrations, and emissions factors were meticulously vetted and their quality assured; they were subsequently published in esteemed academic journals, and coefficients of variations were from Liu *et al*., Niu *et al*., and Peng *et al*.^24–26^. Finally, regarding straw field management, cities lacking data on wheat grain yield per hectare and planted acreage were annotated as “NA” in data records and “blank” on maps to provide context and reduce estimation uncertainty.

Before Monte Carlo procedure was performed, we checked the data distribution of all variables by QQ-plot and found that the normal distribution conformed to its data distribution law. The Monte Carlo procedure was performed for straw nutrient concentrations input, and gaseous pollutant emissions, using normal distributions with the parameters described above and repeated 1,000 times. We report uncertainty from the Monte Carlo model as the 5th-95th percentiles of the resulting flux distribution. The distribution of uncertainty for straw nutrient concentrations input, and gaseous pollutant emissions was described using standard deviations (see Supplement Figure S1 and S2).

### Comparison with the existing studies

**Fig. 7:**
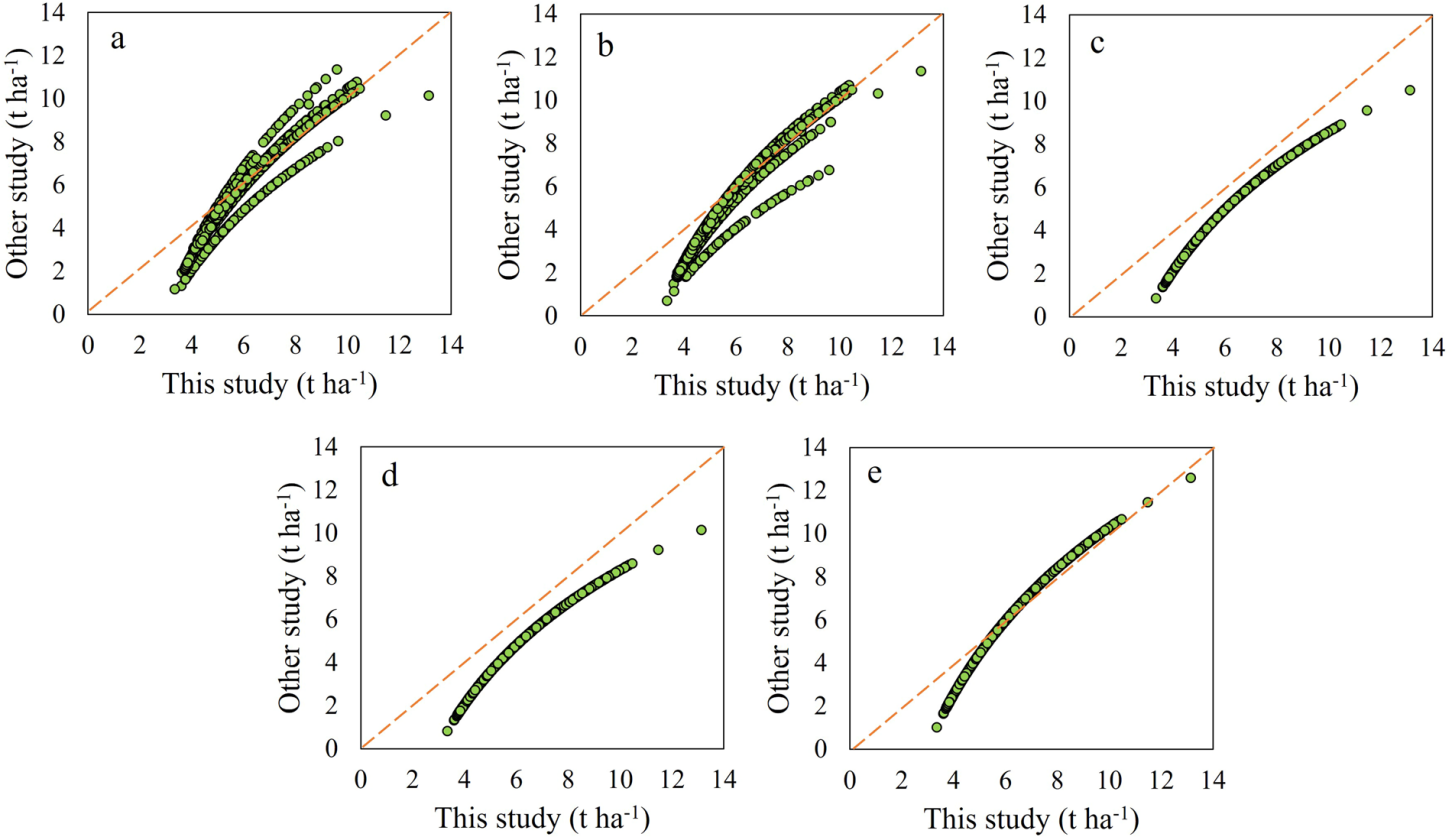
The comparison of the straw-to-grain ratio results with the existing studies. The study for (**a**–**e**) are derived from Liu *et al*.^24^, office data^31^, Li *et al*.^29^, Zhang *et al*.^30^, and public book^32^,respectively.

The accuracy of estimating crop straw yield depends heavily on the ratio of straw-to-grain yield. In view of the fact that the current ratio of straw to grain is a constant coefficient, our study recalculates the mathematical relationship between grain yield and straw yield based on the mathematical relationship between grain yield and harvest index (Fig. 2b), indicating the exponential model could more accurately show the mathematical relationship between them (Fig. 2a). There are many researches focusing on straw-to-grain ratio, including meta-analysis^24,29,30^, and official data^31,32^. We compare our research straw yield calculated by exponential model with relevant study as shown in Fig. 7 and Table S3. The scatters are distributed near the 1:1 diagonal line, which proves the similarity of our data with the existing research. Liu *et al*.^24^ demonstrated 6 regional straw-to-grain ratio by summing up 301 academic papers and 4,995 observations, which underestimated the straw yield compared to this study (Fig. 7a). We also compared the data of straw yield with official data^31^, which similarly underestimated the straw yield calculated d by 6 regional straw-to-grain ratio (Fig. 7b). Li *et al*.^29^ and Zhang *et al*.^30^ also showed the straw-to-grain ratio by summing up 6 academic papers and 839 observations, and 3 public books and 4 academic papers, which was lower than our estimate (Fig. 7c,d). A public White Paper^32^ published by office also summed up the straw-to-grain ratio, which also underestimated the straw yield in comparison to this study (Fig. 7e). We believe these variations are acceptable considering the differences between the studies in methodologies and these scatters are very close to the 1:1 diagonal line.

This study’s dataset, which essentially covers all wheat ecological zones from 2000 to 2021, representing China’s most recent trends in wheat production and dry matter distribution. The scientific merit and novelty of the study is to establish the new regional empirical model of straw yield based on understanding the carbohydrate partitioning pattern. The development of straw yield models would also help with precise assessments of the relative environmental costs of different straw management regimes in wheat-based agricultural systems, from wheat grain production to straw management. We believe that estimating straw yield in various regions could be more accurate by using the exponential model. Meanwhile, this study had certain deficiencies, which only considered the three geographical regions and ignored the Chinese ecological wheat planting zones. In the future, it is necessary to establish the exponential model between grain yield and straw yield in Chinese ecological wheat planting zones such that straw yield can be estimated more accurately. This conceptual framework could serve as a reference for simultaneously ensuring agricultural and environmental security apart from China and wheat agricultural systems.

We also compared the straw nutrient concentration used in this study with other existing studies, and the relevant results were shown in Table 1. To estimate the nutrient supply potential from straw recycling and burning, we used the measured regional wheat straw nutrient concentration in the three regional ecological wheat planting zones, reported by Niu *et al*.^25^. Liu *et al*.^24^ and Li *et al*.^29^ also summed up 8,281 and 152 observations to obtain the constant wheat straw nutrient concentration. Tan *et al*.^33^ determined the straw nutrient content of 13 years wheat localization experiments in northern China and central China.

**Table 1:** The comparison of straw nutrient concentrition used in this study with the existing studies (%).

	This study^25^	Relevant studies^24,29,33^
N	NC: 0.49 CC: 0.52 SC: 0.51	0.54^24^
		0.64^29^
		NC: 0.74^33^ CC: 0.46^33^
P	NC: 0.032 CC: 0.037 SC: 0.032	0.09^24^
		0.12^29^
		NC: 0.081^33^ CC: 0.038^33^
K	NC: 1.801 CC: 1.809 SC: 1.801	1.16^24^
		1.27^29^
		NC: 2.53^33^ CC: 1.83^33^

NC, CC, and SC represent northern China, central China, and southern China, respectively.

The comparison of gaseous pollutant emissions factors resulting from straw burning used in this study with the existing studies was shown in Table 2. To estimate the gaseous pollutant emissions resulting from straw burning, we compared the various emissions factors from the existing studies, and eventually adopted emissions factors reported by Peng *et al*.^26^. This research^26^ summed up 7 academic papers including meta-analysis and actual measurements, and we considered the emissions factors were more representative than other studies. The relevant emissions factors were not for wheat but rather for the three crops (wheat, maize, and rice), as summarized by Lu *et al*.^34^. With the exception of Wang *et al*.^35^, the partial emissions factors based on the 10, 11, and 10 academic papers were summarized by Wang *et al*.^35^, Tian *et al*.^36^, and Zhu *et al*.^37^. Cao *et al*.^38^ determined the partial emissions factors by 8 actual measurements.

**Table 2:** The comparison of gaseous pollutant emissions factors resulting from straw burning used in this study with the existing studies (g kg^−1^).

	This study^26^	Relevant studies^34–38^
PM_2.5_	7.60	9.65^34^
		20.07^35^
		6.37^36^
		3.90^37^
		9.64^38^
SO_2_	0.85	0.54^34^
		0.56^35^
		0.37^36^
		0.40^37^
		0.049^38^
NOx	3.30	3.80^34^
		3.37^35^
		2.55^36^
		2.50^37^
		2.59^38^
NH_3_	0.37	0.52^34^
		0.78^35^
		—
		—
		—
CH_4_	3.4	3.90^34^
		3.50^35^
		3.89^36^
		—
		—
CO_2_	1460	1410^34^
		1445^35^
		1446^36^
		1484^38^
		—

### Supplementary information

Table

## Data Availability

No specific code was used to produce the data described in this manuscript.
