# Supplementary material for: Up-bottom assessments of nutrient supply and gaseous pollutant from Chinese wheat straw field management
Source: Sci Data. 2024 Apr 3;11:329. doi: 10.1038/s41597-024-03151-0 (PMC10991304; doi:10.1038/s41597-024-03151-0)
Supplement: Supplementary file 1 — Table [file 41597_2024_3151_MOESM1_ESM.pdf]

Supplementary Information of
Up-bottom assessments of nutrient supply and gaseous pollutant from Chinese wheat straw field management

Dongxue Li[1,2], Jun Gu[1,2], Xiaoqin Chen[1,2], Yiliu Wang[1,2], Dianjun Lu[1,2]✉, Solomon Yokamo[1,2], Huoyan Wang[1,2] & Peng Hou[3]✉

1. State Key Laboratory of Soil and Sustainable Agriculture, Institute of Soil Science, Chinese Academy of Sciences, Nanjing, 210008, China
2. College of Modern Agricultural Sciences, Chinese Academy of Sciences, Beijing, 100049, China
3. Institute of Crop Sciences/Key Laboratory of Crop Physiology and Ecology, Chinese Academy of Agricultural Sciences, Ministry of Agriculture and Rural Affairs, Beijing 100081, China.
corresponding authors: Dianjun Lu (djlu@issas.ac.cn). Peng Hou (houpeng@caas.cn).

The Supplementary Information includes:

Table S1 **Wheat planting acreage and grain yield per hectare at the province level (Mean ± SE)** ······Page 2

Table S2 **The proportional data regarding straw recycling, straw burning, and straw removing from 2011 to 2015 in mainland China.** ······Page 3

Table S3 **The comparison of the straw to grain ratio results with the existing studies**······Page 4

Figure S1. **Uncertainties of city-level spatial distribution of nutrient supply from straw recycling and burning, detailing (a) nitrogen (N), (b) phosphorus (P), and (c) potassium (K) from 2011-2015 in China**······Page 5

Figure S2. **Uncertainties of spatial visualization of gaseous pollutants resulting from straw burning at the city level in China from 2011–2015. Emissions include (a) $PM_{2.5}$, (b) SO_2, (c) NOx, (d) NH_3, (e) CH_4, and (f) CO_2**······Page 6

Source file of “Empirical Model of Wheat Straw Yield” database······Page 7

Table S1 **Wheat planting acreage and grain yield per hectare at the province level (Mean ± SE) from 2011 to 2015 in mainland China.**

Province/Municipality	Planting acreage (1000 ha)	Grain yield (kg/ha)
Anhui	157±1.9	4964±20.8
Fujian	0±0	948±30.3
Gansu	57±0.6	4250±25.1
Guangdong	0±0	0±0
Guangxi	0±0	0±0
Guizhou	28±0.4	1985±6.8
Hainan	0±0	0±0
Hebei	209±3.0	5236±46.4
Heilongjiang	18±0.8	2450±29.5
Henan	299±2.3	6128±13.0
Hubei	72±1.0	3149±12.1
Hunan	0±0	0±0
Inner Mongolia	243±3.5	4027±91.9
Jiangsu	180±1.8	5536±5.4
Jiangxi	1±0.2	1798±96.4
Jinlin	0±0	0±0
Liaoning	0.4±0	2838±35.0
Ningxia	31±0.8	3682±41.8
Qinghai	10±0.3	3853±34.6
Shaanxi	92±2.1	3467±23.8
Shandong	233±1.8	6169±7.0
Shanxi	60±2.0	3686±32.4
Sichuan	28±0.4	3661±5.0
Tibet	8±0	6538±0
Xinjiang	71±1.0	5114±20.1
Yunnan	25±0.2	2263±7.6
Zhejiang	10±0.2	2920±28.9
Beijing	38±3.3	5169±35.4
Chongqing	106±5.6	3130±18.3
Hong Kong	NA	NA
Macao	NA	NA
Shanghai	NA	NA
Taiwan	NA	NA
Tianjin	111±0.4	5363±46.9

Table S2 **The proportional data regarding straw recycling, straw burning, and straw removing from 2011 to 2015 in mainland China.** The data presented do not include provinces with zero wheat acreage and no available data.

Province/Municipality	Straw recycling (%)	Straw burning (%)	Straw removing (%)
Anhui	57.21	20.9	21.89
Gansu	46.00	13.88	40.12
Guizhou	40.55	31.01	28.44
Hebei	89.98	8.38	1.64
Heilongjiang	97.00	0	3.00
Henan	79.47	8.38	12.15
Hubei	64.31	32.38	3.31
Hunan	51.92	0	48.08
Inner Mongolia	46.00	13.88	40.12
Jiangsu	67.90	6.16	25.94
Jiangxi	67.90	6.16	25.94
Jinlin	97.00	0	3.00
Liaoning	97.00	0	3.00
Ningxia	46.00	13.88	40.12
Qinghai	46.00	13.88	40.12
Shaanxi	46.00	13.88	40.12
Shandong	83.68	8.38	7.94
Shanxi	90.00	8.38	1.62
Sichuan	40.55	31.01	28.44
Tibet	40.55	31.01	28.44
Xinjiang	46.00	13.88	40.12
Yunnan	40.55	31.01	28.44
Zhejiang	100.00	0	0
Beijing	89.98	8.38	1.64
Chongqing	40.55	31.01	28.44
Shanghai	88.00	0	12.00
Tianjin	89.98	8.38	1.64

Table S3 **The comparison of the straw-to-grain ratio results with the existing studies.** NE, N, MLYR, NW, SW, and SE represent Northeast, North, Middle and lower Yangtze River, Northwest, Southwest, and Southeast, respectively.

Sources	Straw-to-grain ratio
This study	Northern China: $y = 2.9616e^{0.1615x}$<br>Central China: $y = 2.4265e^{0.1444x}$<br>Southern China: $y = 1.7096e^{0.2091x}$
Liu et al.[1]	NE: 1.56<br>N: 1.34<br>MLYR: 1.39<br>NW: 1.10<br>SW: 1.49<br>SE: 1.36
Office data[2]	NE: 0.93<br>N: 1.34<br>MLYR: 1.38<br>NW: 1.23<br>SW: 1.31<br>SE: 1.38
Li et al.[3]	1.14
Zhang et al.[4]	1.10
Public book[5]	1.366

Note: x, and y represented crop grain yield, and straw yield, respectively.

Reference:

1. Liu, X. Study on nutrients balance and requirement in agricultural production in China. Chinese Acad Agric Sci (2018).

2. Rural energy and environment agency, ministry of agriculture and rural affairs. Notice on issuing <Technical Guidelines for the Full Treatment and Utilization of Regional Crop Straw> (2017-02-24). http://www.reea.agri.cn/sttzgg/201702/t20170224_5494334.htm (2017).

3. Li, T. et al. Nutrient resource quantity from main grain crop straw incorporation and its enlightenment on chemical fertilizer reduction in wheat production in China. Scientia Agricultura Sinica 53(23): 4835-4854 (2020).

4. Zhang, Q. et al. Re-estimation of direct nitrous oxide emission from agricultural soils of China via revised IPCC 2006 guideline method. Chinese journal of Eco-Agriculture 18(1): 7-13 (2010).

5. Department of basic industry development, state development planning commission of the People's Republic of China. China New Energy and Renewable Energy 1999 White Paper. China Planning Press, 2000.

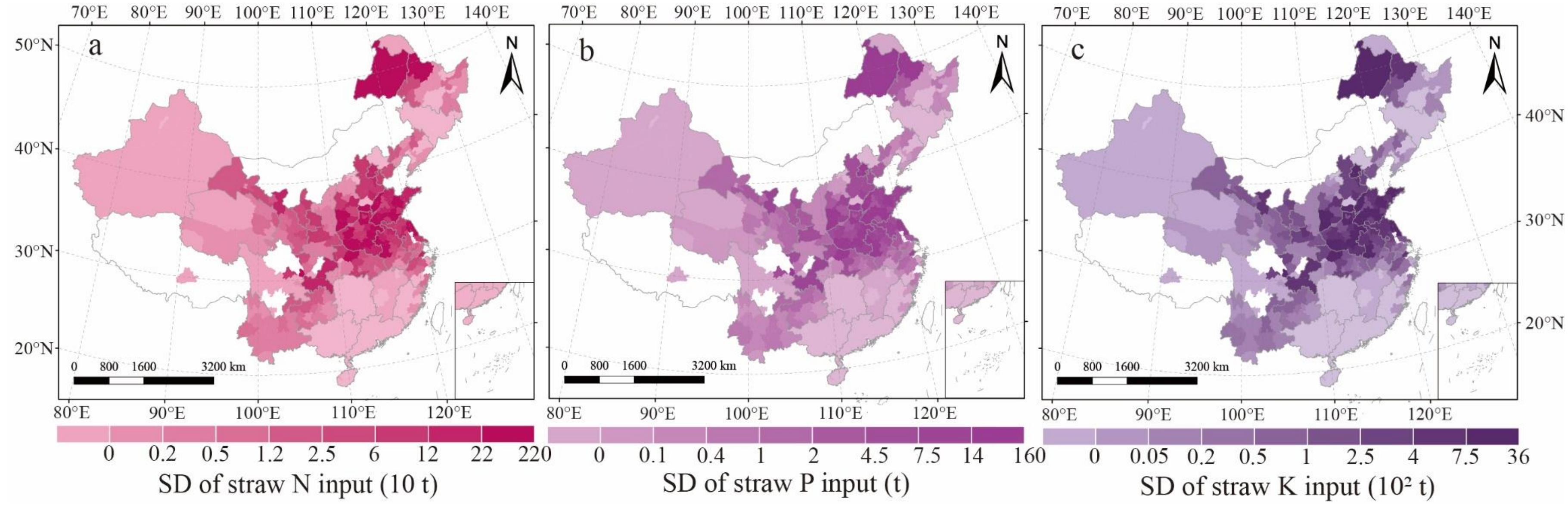

Figure S1. **Uncertainties of city-level spatial distribution of nutrient supply from straw recycling and burning, detailing (a) nitrogen (N), (b) phosphorus (P), and (c) potassium (K) from 2011-2015 in China.** Map of the standard deviation (SD) of the straw nutrient supply.

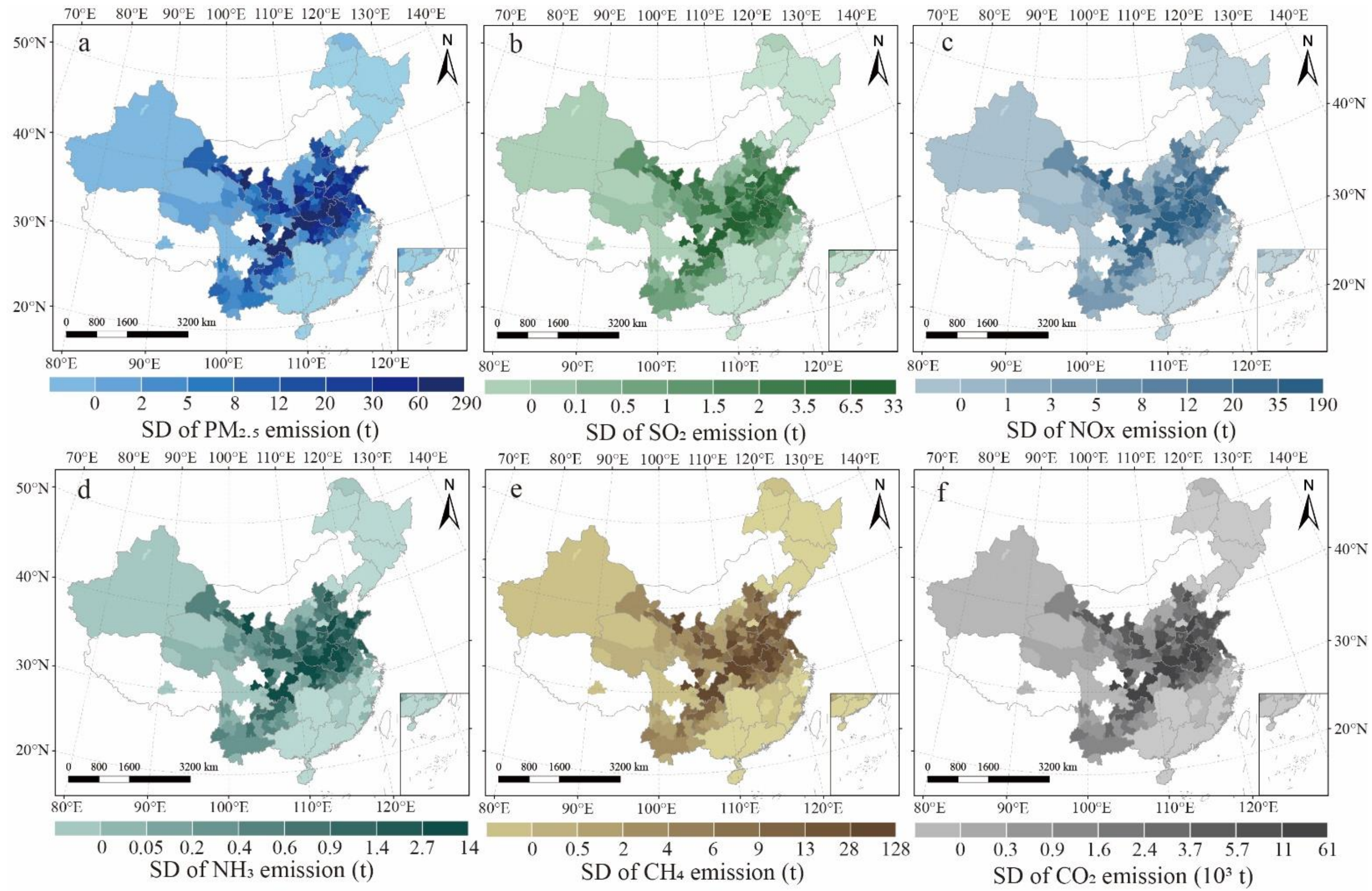

Figure S2. **Uncertainties of spatial visualization of gaseous pollutants resulting from straw burning at the city level in China from 2011–2015. Emissions include (a) $PM_{2.5}$, (b) SO_2, (c) NOx, (d) NH_3, (e) CH_4, and (f) CO_2.** Map of the standard deviation (SD) of the gaseous pollutants.

Source file: The file contains the information of relevant literatures used for extracting data to develop “Empirical Model of Wheat Straw Yield” database across diverse regions of for northern China (NC), central China (CC), and southern China (SC), respectively.

The relevant literatures of NC.

Code	Literatures	Observations
1	Lai, X. Effects of Precipitation Variation on Productivity, Water and Nitrogen Use of Annual Forage - Winter Wheat Rotation Systems on the Loess Plateau. PhD thesis, Lanzhou University, 2022. (in Chinese with English abstract)	48
2	Wang, J. et al. Effects of straw returning instead of fertilizer on wheat yield and water and fertilizer utilization in Loess Dryland. *Journal of Soil and Water Conservation* **36**, 236-243 (2022). (in Chinese with English abstract)	16
3	Ren, G. Effects of different straw returning and tillage methods on dry matter accumulation and yield of wheat. *Special Economic Animals and Plants* **26**, 54-56 (2023). (in Chinese with English abstract)	6
4	Qi, S. Effect of phosphorus fertilizer varieties on crop yield and phosphorus in Eum-Orthic Anthrosols. Master thesis, Northwest A&F University, 2022. (in Chinese with English abstract)	17
5	Li, Y. Effects of Mulching Methods on Soil Carbon Emission and Plough Layer Environment of Farmland in Dryland. PhD thesis, Gansu Agricultural University, 2022. (in Chinese with English abstract)	32
6	Wang, J. Effects of straw incorporation replacing fertilizer on Winter Wheat Yield and environmental and economic benefits in Loess Dryland. Master thesis, Shanxi Agricultural University, 2022. (in Chinese with English abstract)	16
7	Du, W. Effects of water and fertilizer reduction and straw returning on crop yield and nitrogen and phosphorus efficiency of wheat-maize system. Master thesis, Northwest A&F University, 2022. (in Chinese with English abstract)	15
8	Li, G. Study on the technology of reducing phosphorus and increasing efficiency of wheat-maize crop system in calcareous soil of Guan Zhong. Master thesis, Northwest A&F University, 2021. (in Chinese with English abstract)	14
9	Liu, W. Effects of Corn Straw Returning and Supporting Measures on Wheat Yield and Soil Properties. Master thesis, Northwest A&F University, 2020. (in Chinese with English abstract)	84
10	Liu, W. Responses of Dryland Winter Wheat Yield Formation and Soil Carbon and Nitrogen Pools to Topdressing under Straw Mulching. Master thesis, Northwest University, 2020. (in Chinese with English abstract)	28
11	Hu, C. Effects and mechanisms of pre-sowing soil water, nitrogen and population size on winter wheat yield and water use efficiency under	168

	straw mulching. PhD thesis, Northwest A&F University, 2020. (in Chinese with English abstract)	
12	Hou, Y. Effects of different fertilization treatments on Yield and nutrient absorption of Winter Wheat in Shaya County. Master thesis, Xinjiang Agricultural University, 2020. (in Chinese with English abstract)	15
13	Lei, J. Effect of drip irrigation layout, water and nitrogen supply on yield and quality formation of winter wheat in Xinjiang. PhD thesis, China Agricultural University, 2017. (in Chinese with English abstract)	22
14	Chen, Y. Effects of different sowing patterns on Growth and yield of winter wheat with drip irrigation. Master thesis, Xinjiang Agricultural University, 2016. (in Chinese with English abstract)	6
15	Liu, P. Effect of seeding rate and planting density on population trait and grain yield of drip irrigated wheat. Master thesis, Shihezi University, 2012. (in Chinese with English abstract)	30
16	Meng, F. Effect of tillage on wheat yield and soil quality in arid areas. Master thesis, Xinjiang Agricultural University, 2007. (in Chinese with English abstract)	4
17	Miao, F. Effects of soil structure, water and crop growth under different tillage depths. Master thesis, Ningxia University, 2021. (in Chinese with English abstract)	5
18	Hao, W. Effects of continuous reduction of chemical fertilizer on growth, yield and quality of spring wheat. Master thesis, Ningxia University, 2022. (in Chinese with English abstract)	10
19	Li, C. Limitations and Its Regulations for Improving Wheat Grain Yield, Nutritional Quality, and Fertilizer Use Efficiency in Drylands of the Loess Plateau. PhD thesis, Northwest A&F University, 2020. (in Chinese with English abstract)	27
20	Chen, Y. Effects of fertilizer application on yield, nutrient uptake and accumulation under the winter wheat/summer maize rotation system in Guanzhong area of Shaanxi. Master thesis, Northwest A&F University, 2012. (in Chinese with English abstract)	10
21	Zan, Y. Effect of nitrogen and phosphorus fertilizer rate on yield, nutrient utilization and grain mineral nutrient quality of wheat in dryland. PhD thesis, Northwest A&F University, 2012. (in Chinese with English abstract)	27
22	Fan, H. Study on succession of varietal character and nutritive peculiarity of wheat in Shaanxi. PhD thesis, Northwest A&F University, 2007. (in Chinese with English abstract)	24
23	Mu, W. Differences in Yield, Grain Sulfur Concentration and Sulfur Requirement of Wheat Cultivars in Main Wheat Production Regions of China. Master thesis, Northwest A&F University, 2022. (in Chinese with English abstract)	7
24	Wang, S. Effects of combined application of organic and inorganic fertilizer on yield and nutrient uptake and utilization of winter wheat in	10

	dryland. Master thesis, Northwest A&F University, 2022. (in Chinese with English abstract)	
25	Li, W. Root pruning affects winter wheat performance and phosphorus uptake. Master thesis, Northwest A&F University, 2022. (in Chinese with English abstract)	11
26	Lian, H. Response of yield and water and nitrogen utilization efficiency to different nitrogen levels in dryland winter wheat (*Triticum aestivum L.*) released in different decades of Shaanxi Province of China. PhD thesis, Northwest A&F University, 2020. (in Chinese with English abstract)	50
27	Zhang, D. The regulatory effect of root pruning on winter wheat yield and phosphorus absorption at different phosphorus levels. Master thesis, Northwest A&F University, 2020. (in Chinese with English abstract)	18
28	Xu, J. Effects of Plastic Film Mulch and Nitrogen Application Rate on Winter Wheat Yield, Water and Nitrogen Use Efficiency. Master thesis, Northwest A&F University, 2020. (in Chinese with English abstract)	10
29	Xiang, D. The relationship between cultivar-plant competition and optimal population yield on Northern Winter Wheat Regions. Master thesis, Lanzhou University, 2019. (in Chinese with English abstract)	24
30	Zhang, J. Wheat-maize anniversary in Yuncheng district technical research of water and fertilizer. Master thesis, Shanxi Agricultural University, 2018. (in Chinese with English abstract)	7
31	Li, N. Effects of water and temperature in winter wheat and interactions of nitrogen and sulfur in summer maize and their optimal regulation. PhD thesis, Northwest A&F University, 2018. (in Chinese with English abstract)	12
32	Diao, C. Difference in grain nitrogen and phosphorus contents of high-yielding wheat cultivars and its relation to yield formation and nutrients uptake and utilization in drylands. Master thesis, Northwest A&F University, 2018. (in Chinese with English abstract)	32
33	Gao, F. Effect of variety, density, water and fertilizer amount on yield, the absorption and utilization of water and nutrient of wheat-maize. Master thesis, Northwest A&F University, 2018. (in Chinese with English abstract)	27
34	Liu, Y. Effects of applying organic fertilizer and zinc fertilizers on wheat growth and utilization of water and fertilizer. Master thesis, Northwest A&F University, 2018. (in Chinese with English abstract)	24
35	Li, S. Differences in grain zinc concentrations of high-yielding wheat cultivars and its relation to yield formation and nutrients uptake and utilization in drylands. Master thesis, Northwest A&F University, 2018. (in Chinese with English abstract)	16
36	Yao, D. Effect of water and fertilizer on maize-wheat rotation based on the method of “3414”. Master thesis, Northwest A&F University, 2018. (in Chinese with English abstract)	79

37	Wang, L. Nitrogen and manure fertilizers regulate the water and nitrogen use of winter wheat in dryland. PhD thesis, Northwest A&F University, 2018. (in Chinese with English abstract)	34
38	Huang, M. Optimized fertilization for cutting down fertilizers and increasing efficiency of wheat production based on soil test at harvest and regulating fertilizer placement in dryland. PhD thesis, Northwest A&F University, 2017. (in Chinese with English abstract)	77
39	Cui, T. The grain yield and stable analysis of the spring wheat in semiarid environment. Master thesis, Lanzhou University, 2017. (in Chinese with English abstract)	54
40	She, X. Reasons for wheat grain zinc difference among fields in dryland areas. Master thesis, Northwest A&F University, 2017. (in Chinese with English abstract)	3
41	Wang, J. Evolvement of physio-ecological characteristics and mechanism of yield formation in dryland wheat. PhD thesis, Lanzhou University, 2016. (in Chinese with English abstract)	84
42	Wang, X. Study on high yield and high quality of deep tillage in fallow period and nitrogen and phosphorus fertilizer of dryland wheat. Master thesis, Shanxi Agricultural University, 2016. (in Chinese with English abstract)	6
43	Han, X. Responses of grain yield and nitrogen use efficiency of wheat cultivars released in different decades to soil fertility in Shaanxi Guanzhong plain. Master thesis, Northwest A&F University, 2016. (in Chinese with English abstract)	43
44	Wang, F. Effects of the introduction of Dwarf Genes on water use efficiency of winter wheat. Master thesis, Northwest A&F University, 2016. (in Chinese with English abstract)	14
45	Sun, Y. Responding mechanism of ecosystem carbon sequestration benefits to vegetation restoration on the loess plateau of China. PhD thesis, Northwest A&F University, 2015. (in Chinese with English abstract)	73
46	Jin, Z. Effect of organic manure application combined with different rates nitrogen fertilizer in yield, water and nitrogen utilization of winter wheat and soil nutrition on Weibei dryland. Master thesis, Northwest A&F University, 2014. (in Chinese with English abstract)	20
47	Liu, J. Effects of population size and nitrogen application on winter wheat grain yield and water use under straw mulching on dryland. Master thesis, Northwest A&F University, 2014. (in Chinese with English abstract)	15
48	Li, M. Responses of winter wheat to zinc fertilization and soil moisture. Master thesis, Northwest A&F University, 2013. (in Chinese with English abstract)	39
49	Li, H. Grain yield formation and nutrient use of winter wheat under soil surface mulching on dryland. PhD thesis, Northwest A&F University,	9

	2012. (in Chinese with English abstract)	
50	Wang, J. Effects of selenium and zinc on yield and mineral nutrition of main crops on dryland. PhD thesis, Northwest A&F University, 2012. (in Chinese with English abstract)	58
51	Xue, C. Soil plant nutrition basics for high yield and high water and nutrient use efficiency cultivation of winter wheat on dryland in northwest of China. Master thesis, Northwest A&F University, 2011. (in Chinese with English abstract)	12
52	Meng, X. Effect of soil moisture before sowing and nitrogen and phosphorus rates on dryland winter wheat yield formation, nutrient and water use. Master thesis, Northwest A&F University, 2011. (in Chinese with English abstract)	20
53	Zhou, L. Nutritional and agronomic mechanism for high yield and high efficiency production of winter wheat under low nutrient input condition in dryland area. Master thesis, Northwest A&F University, 2010. (in Chinese with English abstract)	30
54	Zhao, X. Nitrogen efficiency and the effect of application of N fertilizer on N, P and K uptake and translocation of wheat. Master thesis, Northwest A&F University, 2010. (in Chinese with English abstract)	10
55	Zheng, X. Study on the transport and distribution of crop nutrients and its regulation in dryland crops with different cultivated systems. PhD thesis, Northwest A&F University, 2006. (in Chinese with English abstract)	71
56	Kang, K. Studies on photosynthetic property during evolutional succession of spring wheat varieties in irrigation district of Ningxia. Master thesis, Ningxia University, 2004. (in Chinese with English abstract)	8
57	Chai, Y. Phenotyping analysis of wheat based on CTD, SRI and GWAS of some key traits. PhD thesis, Northwest A&F University, 2022. (in Chinese with English abstract)	12
58	Li, Y. Effects of Mulching Methods on Soil Carbon Emission and Plough Layer Environment of Farmland in Dryland. PhD thesis, Gansu Agricultural University, 2022. (in Chinese with English abstract)	33
59	Wu, S. Effects of Nitrogen Reduction and Biochar Application on Wheat Productivity and Soil Quality in Dryland. Master thesis, Northwest A&F University, 2022. (in Chinese with English abstract)	22
60	Li, R. Regulation of mulching on ecophysiological characteristics and water consumption of winter wheat in dryland. PhD thesis, Gansu Agricultural University, 2022. (in Chinese with English abstract)	30
Total	---	1728

The relevant literatures of CC.

Code	Literatures	Observations
1	Lu, D. et al. Manure Limits Wheat Yield Losses Due to Delayed Seeding.	63

	Agron. J. **107**, 2294-2302 (2015).	
2	Pan, J. et al. Long-term optimization of crop yield while concurrently improving soil quality. *Land Degrad Dev.* **30**, 897-909 (2019).	32
3	Lu, D. Dynamics of population trait for high yielding and high efficiency winter wheat and N nutrient regulation in the North China Plain. PhD thesis, China Agricultural University, 2015. (in Chinese with English abstract)	18
4	Meng, Q. et al. Alternative cropping systems for sustainable water and nitrogen use in the North China Plain. *Agric. Ecosyst. Environ.* **146**, 90-102 (2012).	24
5	Yue, S. et al. Critical nitrogen dilution curve for optimizing N management of winter wheat production in the North China Plain. *Agron. J.* **104**, 523-529 (2012).	60
6	Lu, D. et al. Elucidating population establishment associated with N management and cultivars for wheat production in China. *Field Crop. Res.* **163**, 81-89 (2014).	40
7	Lu, D. et al. The effects of cultivar and nitrogen management on wheat yield and nitrogen use efficiency in the North China Plain. *Field Crop. Res.* **171**, 157-164 (2015).	40
8	Guo, J. The design and validation of high yield technologies for winter wheat in the ecological condition of Quzhou county. Master thesis, China Agricultural University, 2011. (in Chinese with English abstract)	42
9	Ma, Y. et al. Effect of Nitrogen Application on Dry Matter Accumulation, Yield and Nitrogen Utilization Efficiency of Winter Wheat. *Acta Agriculturae Boreali-Sinica* **28**, 187-192 (2013). (in Chinese with English abstract)	111
10	Sun, J. et al. Effects of Different Management Modes on Yield and Nutrients Absorption of Winter Wheat. *Journal of Anhui Agri. Sci.* **40**, 12833-12835 (2012). (in Chinese with English abstract)	60
11	Ye, Y. et al. Effect of nitrogen application on dry matter accumulation and translocation in wheat. *Journal of Triticeae Crops* **32**, 488-493 (2012). (in Chinese with English abstract)	90
12	Lu, D. et al. Integrated crop-N system management to establish high wheat yield population. *Field Crop. Res.* **191**, 66-74 (2016).	24
13	Ye, Y. et al. Understanding physiological processes associated with yield-trait relationships in modern wheat varieties. *Field Crop. Res.* **124**, 316-322 (2011).	48
14	Xu, Z. Potassium uptake by rotated wheat-maize crops and the effects of potassium on stalk strength. Master thesis, China Agricultural University, 2016. (in Chinese with English abstract)	136
15	Deng, Y. et al. Arbuscular mycorrhizal fungal colonization is considerable at optimal Olsen-P levels for maximized yields in an intensive wheat-maize cropping system. *Field Crop. Res.* **209**, 1-9 (2017).	48

16	Zhang, W. et al. Overuse of Phosphorus Fertilizer Reduces the Grain and Flour Protein Contents and Zinc Bioavailability of Winter Wheat (Triticum aestivum L.). *J. Agric. Food Chem.* **65**, 1473-1482 (2017).	48
17	Cui, Z. et al. In-season nitrogen management strategy for winter wheat: Maximizing yields, minimizing environmental impact in an over-fertilization context. *Field Crop. Res.* **116**, 140-146 (2010).	48
18	Cui, Z. et al. Regional Evaluation of Critical Nitrogen Concentrations in Winter Wheat Production of the North China Plain. *Agron. J.* **101**, 159-166 (2009).	48
19	Cui, Z. et al. On-Farm Evaluation of Winter Wheat Yield Response to Residual Soil Nitrate-N in North China Plain. *Agron. J.* **100**, 1527-1534 (2008).	48
20	Cui, Z. et al. Effects of N fertilization on winter wheat grain yield and its crude protein content and apparent N losses. *The journal of Applied Ecology*, **11**, 67-71 (2005). (in Chinese with English abstract)	48
21	Cui, Z. et al. Effect of N Fertilization on Grain Yield of Winter Wheat and Apparent N Losses. *Pedosphere* **16**, 806-812 (2006).	48
22	Cui, Z. et al. Using In-Season Nitrogen Management and Wheat Cultivars to Improve Nitrogen Use Efficiency. *Soil Sci. Soc. Am. J.* **75**, 976-983 (2011).	48
23	Cui, Z. et al. On-farm estimation of indigenous nitrogen supply for site-specific nitrogen management in the North China plain. *Nutr. Cycl. Agroecosyst.* **81**, 37-47 (2008).	24
24	Cui, Z. et al. On-farm evaluation of an in-season nitrogen management strategy based on soil N_{min} test. *Field Crop. Res.* **105**, 48-55 (2008).	33
25	Cui, Z. et al. Development of Regional Nitrogen Rate Guidelines for Intensive Cropping Systems in China. *Agron. J.* **105**, 1411-1416 (2013).	48
26	Yue, S. et al. Change in Nitrogen Requirement with Increasing Grain Yield for Winter Wheat. *Agron. J.* **104**, 1687-1693 (2012).	48
27	Meng, Q. Strategies for achieving high yield and high nutrient use efficiency simultaneously for maize (*Zea mays L.*) and wheat (*Triticum aestivum L.*). PhD thesis, China Agricultural University, 2012. (in Chinese with English abstract)	50
28	Liu, Z. et al. Yield Gap Analysis of County Level Irrigated Wheat in Hebei Province, China. *Agron. J.* **111**, 2245-2254 (2019).	57
29	Chen, X. et al. Producing more grain with lower environmental costs. *Nature* **514**, 486-489 (2014).	49
30	Ju, X. et al. Evaluation of Nitrogen Loss Way in Winter Wheat and Summer Maize Rotation System. *Scientia Agricultura Sinica* **12**, 1493-1499 (2002). (in Chinese with English abstract)	48
31	Liu, X. et al. Nitrogen recommendation for winter wheat using N_{min} test and rapid plant tests in North China Plain. *Commun. Soil Sci. Plant Anal.* **34**, 2539-2551 (2003).	52
32	Zou, G. et al. Study on Soil Denitrification in Winter Wheat-Summer	50

	Maize Rotation System. *Scientia Agricultura Sinica* **37**, 1492-1496 (2004). (in Chinese with English abstract)	
33	Liu, X. et al. Effect of reduced N application on N utilization and balance in winter wheat-summer maize cropping system. *Chinese Journal of Applied Ecology* **15**, 458-462 (2004). (in Chinese with English abstract)	50
34	Ju, X. et al. Soil nitrogen mineralization and its prediction in winter wheat-summer maize rotation system. *Chinese Journal of Applied Ecology* **14**, 2241-2245 (2003). (in Chinese with English abstract)	45
35	Ju, X. et al. The fate of nitrogen fertilizer in winter wheat growth season under high soil fertility condition. *Acta Agriclturae Nucleatae Sinica* **16**, 397-402 (2002). (in Chinese with English abstract)	48
36	Ju, X. et al. Accumulation and movement of NO_3-N in soil profile in winter wheat-summer maize rotation system. *Acta Pedologica Sinica* **40**, 538-546 (2003). (in Chinese with English abstract)	55
37	Liu, X. et al. Effect of N Application as Basal Fertilizer on Grain Yield of Winter Wheat, Fertilizer N Recovery and N Balance. *Acta Ecologica Sinica* **22**, 1122-1128 (2002). (in Chinese with English abstract)	52
38	Liu, X. et al. Nitrogen dynamics and budgets in a winter wheat–maize cropping system in the North China Plain. *Field Crop. Res.* **83**, 111-124 (2003).	65
39	Ju, X. et al. Study on the fate of nitrogen fertilizer in winter wheat/summer maize rotation system in Beijing suburban. *Plant nutrition and fertilizer science* **9**, 264-270 (2003). (in Chinese with English abstract)	35
40	Ju, X. et al. Study on Effect of Nitrogen Fertilizer and Nitrogen Balance in Winter Wheat and Summer Maize Rotation System. *Acta Agricultura Sinica* **35**, 1361-1368 (2002). (in Chinese with English abstract)	108
41	Zhan, A. et al. Estimating on-farm wheat yield response to potassium and potassium uptake requirement in China. *Field Crop. Res.* **191**, 13-19 (2016).	60
42	Cui, Z. Optimization of the nitrogen fertilizer management for a winter wheat-summer maize rotation system in the north China plain – from field to regional scale. PhD thesis, China Agricultural University, 2005. (in Chinese with English abstract)	60
Total	---	2209

The relevant literatures of SC.

Code	Literatures	Observations
1	Chen, Z. Wheat grain yield, fate of ^{15}N-labeled fertilizer in wheat–soil system, and soil nitrification under root-zone fertilization in Southeast China. PhD thesis, Institute of Soil Science, Chinese Academy of Sciences, 2017. (in Chinese with English abstract)	100
2	Chen, Z. et al. Spatial and temporal nitrogen applications for winter wheat in a loamy soil in south-eastern China. *Nutr. Cycl. Agroecosyst.*	60

	**109**, 43-55 (2017).	
3	Chen, Z. et al. The fates of ^{15}N-labeled fertilizer in a wheat–soil system as influenced by fertilization practice in a loamy soil. *Sci Rep* **6**, 34754 (2016).	149
4	Zhao, X. Evaluation of soil potassium-holding capacity and study on the potassium-holding mechanism. PhD thesis, Institute of Soil Science, Chinese Academy of Sciences, 2019. (in Chinese with English abstract)	144
5	Jiang, S. The factors and effects of root-zone fertilization of phosphorus on rice and wheat. Master thesis, Institute of Soil Science, Chinese Academy of Sciences, 2016. (in Chinese with English abstract)	60
6	Hu, F. The effect of fertilizer placement on the growth of rice and wheat and fertilizer use efficiency. PhD thesis, Institute of Soil Science, Chinese Academy of Sciences, 2016. (in Chinese with English abstract)	72
7	Song, H. Optimization measures of one-time root-zone fertilization technology and mechanism of high yield and high efficiency in wheat and rice. PhD thesis, Institute of Soil Science, Chinese Academy of Sciences, 2023. (in Chinese with English abstract)	118
8	Chen, X. et al. Producing more grain with lower environmental costs. *Nature* **514**, 486-489 (2014).	20
9	Li, C. Study on efficient potassium application strategy for rice - wheat rotation system and its relation to the change of soil potassium fertility in Yangtze river delta. PhD thesis, Institute of Soil Science, Chinese Academy of Sciences, 2020. (in Chinese with English abstract)	335
10	Ding, Y. Characteristics of wheat cultivar with high-yield and -efficiency and its response mechanism of nitrogen. PhD thesis, Yangzhou University, 2022. (in Chinese with English abstract)	126
Total	---	1184
